# Complete genome sequence of *Wolbachia* strain *w*Mel colonizing *Drosophila melanogaster* JW18 cells

**DOI:** 10.1128/mra.00643-25

**Published:** 2025-11-10

**Authors:** Emily M. Layton, Macy T. Vance, Richard W. Hardy, Irene L. G. Newton

**Affiliations:** 1 Department of Biology, Indiana University 1772, Bloomington, Indiana, USA; California State University San Marcos, San Marcos, California, USA

**Keywords:** cell culture, *Wolbachia*, symbiosis, gene loss

## Abstract

*Wolbachia* are widespread insect endosymbionts known for manipulating host reproduction, aiding vector-borne disease control, and influencing host evolution. Here, we provide the complete genome sequence of the *Wolbachia* strain *w*Mel derived from the *Drosophila melanogaster* JW18 cell culture and assembled using a hybrid approach combining Illumina and Oxford Nanopore reads.

## ANNOUNCEMENT

The *Drosophila melanogaster* JW18 cell line (obtained from Dr. Bill Sullivan [UCSC]) colonized with *w*Mel *Wolbachia* is a widely used model in the field for which there is no complete genome. We sought to identify genetic variations that may have been introduced due to long-term adaptation to cell culture.

JW18 cells were maintained in Schneider’s insect medium supplemented with 10% fetal bovine serum and 1% antibiotic-antimycotic solution (penicillin-streptomycin-Gibco Amphotericin B) in unvented culture flasks kept in the dark at room temperature. Cells were scraped from a confluent flask and divided into two aliquots: one for the *Wolbachia* isolation (to increase the proportion of *Wolbachia*-derived reads) and the other for the whole-cell lysate (to preserve longer reads). The *Wolbachia* isolation used a previously published protocol (1) with minor modifications: samples were vortexed in 2 s pulses and spun at 20,000 *g* for 10 min. Both the isolated *Wolbachia* and whole-cell lysate pellets were resuspended in DNA/RNA Shield and kept at room temperature for 30 min before high-molecular weight DNA extraction using the Zymo Quick-DNA HMW MagBead Kit following the manufacturer’s instructions for cells and solids. No pellet was observed after the initial microbial lysis centrifugation step. No shearing or size selection was performed.

The isolated *Wolbachia* sample was sequenced using the Nextera Small Genome DNA Library protocol (LIB-146b) on Illumina NextSeq 2000 with a P2 flow cell (100 cycles, v3 chemistry), generating 918 Mb across 7,522,085 reads (mean length: 61 bp). The whole-cell lysate was sequenced using the Oxford Nanopore Technologies Rapid Barcoding Kit 24 V14 on a MinION Mk1C with an R9.4 flow cell (#FAX48436) and base-called with Dorado v0.5.3, yielding 466,278 reads (N50: 12,413 bp, mean read length: 5,875 bp). The genome was assembled using a hybrid approach, starting with a long-read assembly, followed by short-read polishing (2) with default parameters, unless otherwise specified.

Short-read quality control was performed using fastp v0.24.0. For long-read preprocessing, adaptors were trimmed with Porechop v0.2.4 (3), and reads were filtered with Filtlong v0.2.4 (4) (--min_length 1000, --keep_percent 95). Reads were then mapped to a *D. melanogaster* reference genome (GCA_000001215.4) using minimap2 v2.26-r1175. Unmapped reads totaling 146 Mb across 24,939 reads (N50: 12,415 bp, mean read length: 5,870 bp) were assembled using Autocycler v0.1.2 (5) with no manual intervention. Reads were subsampled into six sets (11,372 reads per subset, ~52.6× depth per subset) and each assembled using Canu v2.2, Flye v2.9.5, miniasm v0.3, NECAT v0.0.1, NextDenovo v2.5.2, and raven v1.8.3 for a total of 36 assemblies. Contigs were clustered, and one cluster passed QC, containing a single >1.2 Mb contig from each assembler. The final consensus assembly produced a circular chromosome (1,274,542 bp, 35.2% GC, ~110× long-read depth). Autocycler detected and trimmed start-end overlaps via self-vs-self overlap alignment of the unitigs. The genome was rotated to the canonical *dnaA* start site with Dnaapler (6–10). The assembly was polished with long reads using medaka v2.0.1 (11) (--bacteria) and with short reads using Polypolish v0.6.0 (12, 13) and Pypolca v0.3.1 (--careful) (13). Annotation with the NCBI Prokaryotic Genome Annotation Pipeline v6.10 identified 1,243 coding sequences, 34 tRNAs, and one copy of each of the 5S, 16S, and 23S rRNA genes. Genome completeness was assessed with CheckM v1.2.4 (99.79% complete, 2.56% contamination, o_Rickettsiales (UID3811) data set) (10, 14–16) and BUSCO v6.0.0 (99.1% complete, rickettsiales odb12 data set) (17).

**Fig 1:**
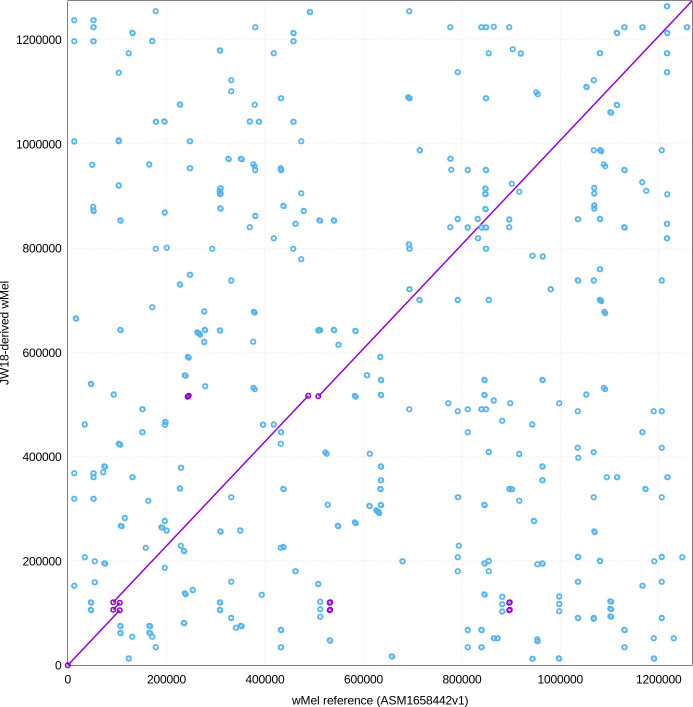
Alignment of the JW18-derived *w*Mel genome with the *w*Mel reference sequence. A NUCmer plot was generated using MUMmer v4.0.1 to visualize genomic rearrangements. Purple lines represent maximal unique matches between the genomes, while blue lines indicate reverse complement matches, with a minimum substring length of 20 bases. Discontinuous lines highlight a triplication and a deletion in the JW18-derived *w*Mel genome relative to the reference.

We identified a ~19.5 kbp deletion corresponding to the previously described Octomom region (18, 19), along with an amplification of a ~14.3 kbp region (threefold increase) containing 12 genes, including those related to metabolite synthesis and export, protein export, a DNA polymerase III subunit, and a DNA gyrase subunit, similar but not identical to a duplication previously found in JW18-derived *w*Mel (20) (Fig. 1).

Structural variation analysis using *breseq* v0.39.0 (21, 22) (--polymorphism-prediction) with both long (--nanopore) and short reads revealed several structural variants (Table 1), raising the intriguing possibility that there are subpopulations of *Wolbachia* with distinct genomic architectures that could underlie differences in phenotypes, consistent with *Wolbachia’s* previously described highly recombining, mosaic genome structure (23).

**TABLE 1:** Predicted SNP and structural variation in wMel colonizing JW18 cells^*a*^

Position	Reads	Score	Freq	Annotation	Gene	Product
= 73311	664 + GAAGGAAG	5/80	72.7%	Coding (405/816 nt)	NEHKPIKF_00076	Hypothetical protein
724915 =				Coding (93/822 nt)	NEHKPIKF_00757	3′−5′ exonuclease
= 321592	17	13/76	9.5%	Intergenic (−66/+706)	NEHKPIKF_00341/NEHKPIKF_00342	Hypothetical protein/hypothetical protein
321794 =				Intergenic (−268/+504)	NEHKPIKF_00341/NEHKPIKF_00342	Hypothetical protein/hypothetical protein
394,635	30	N/A (SNP)	7.50%	D48G (GAC→GGC)	NEHKPIKF_00416	Bcr/CflA family efflux MFS transporter
= 395079	19	10/92	10.6%	Coding (587/1164 nt)	NEHKPIKF_00416	Bcr/CflA family efflux MFS transporter
395477 =				Coding (985/1164 nt)	NEHKPIKF_00416	Bcr/CflA family efflux MFS transporter
766095 =	45 + 24 bp	4/50	24.1%	Coding (+1280/−497)	NEHKPIKF_00795/NEHKPIKF_00796	50S ribosomal protein L9/tRNA-Met
1183286 =				Coding (1142/1230 nt)	NEHKPIKF_01227	Lipoprotein-releasing ABC transporter permease subunit
1178868 =	31	19/92	10.5%	Coding (553/582 nt)	algA_1	Alginate biosynthesis protein AlgA
1191235 =				Coding (1605/2307 nt)	NEHKPIKF_01234	AAA domain-containing protein
= 1179781	17	14/96	6.4%	Coding (28/774 nt)	algA_2	Alginate biosynthesis protein AlgA
= 1191239				Coding (1609/2307 nt)	NEHKPIKF_01234	AAA domain-containing protein

^*a*^ SNP and structural variants were predicted by *breseq* based on the analysis of short reads relative to the JW18-derived *w*Mel consensus assembly and manually curated to exclude poorly supported predictions and extraneous information. New [EL1] junctions are predicted by split reads where each side of the read maps to a different location in the JW18-derived *w*Mel reference genome described here. Paired rows correspond to predicted new junctions; each side of the split read is represented in one row. The “Position” column lists the coordinates in the reference genome that are joined together in the junction, and the equals sign informs how the reads supporting the junction are oriented relative to the reference genome. If each side of the split read is mapped to the reference sequence, “= position” means that starting from the listed position coordinate, the read extends to lower coordinates in the reference genome, whereas “position =” means that starting from the listed position coordinate, the read extends to higher coordinates in the reference genome. The most common type of junction, for example, “= 5” and “11 =”, likely indicates a deletion of bases 6–10. The single row indicates an SNP, with "Position" marking the mutation site. "Reads" specifies the number of supporting reads. "Score" denotes the position-hash (left) and minimum-overlap (right) scores. “Freq” indicates the predicted frequency of that SNP or junction in the population. "Annotation" describes the position of the SNP or junction breakpoint within a gene (coding) or upstream (+) or downstream (−) of the nearest neighboring genes (intergenic). "Gene" lists the affected or flanking genes; "Product" describes their function. See *breseq* output documentation for more details.

## Data Availability

This genome project is available at GenBank under BioProject accession number PRJNA1246252. The complete genome sequence of JW18-derived *w*Mel can be found under GenBank accession number CP186703 and the reported genome is the first version, CP186703.1. The raw reads are available from the NCBI Sequence Read Archive under the accession numbers SRR32971796 for the Illumina reads and SRR32971797 for the Oxford Nanopore FASTQ file.
